# Predictive value of telomere length on outcome following acute myocardial infarction: evidence for contrasting effects of vascular vs. blood oxidative stress

**DOI:** 10.1093/eurheartj/ehx177

**Published:** 2017-04-24

**Authors:** Marios Margaritis, Fabio Sanna, George Lazaros, Ioannis Akoumianakis, Sheena Patel, Alexios S. Antonopoulos, Chloe Duke, Laura Herdman, Costas Psarros, Evangelos K. Oikonomou, Cheerag Shirodaria, Mario Petrou, Rana Sayeed, George Krasopoulos, Regent Lee, Dimitris Tousoulis, Keith M. Channon, Charalambos Antoniades

**Affiliations:** 1 Cardiovascular Medicine Division, University of Oxford, John Radcliffe Hospital, West Wing L6, Headley Way, Oxford OX3 9DU, UK; 2 1st Department of Cardiology, Hippokrateion Hospital, University of Athens, Vas Sofias 114, 11527, Athens, Greece; 3 Department of Cardiac Surgery, Oxford Heart Centre, John Radcliffe Hospital, Headley Way, Oxford OX3 9DU, UK

**Keywords:** Telomere length, Post-procedural outcome, Superoxide, NADPH-oxidases

## Abstract

**Aims:**

Experimental evidence suggests that telomere length (TL) is shortened by oxidative DNA damage, reflecting biological aging. We explore the value of blood (BTL) and vascular TL (VTL) as biomarkers of systemic/vascular oxidative stress in humans and test the clinical predictive value of BTL in acute myocardial infarction (AMI).

**Methods and results:**

In a prospective cohort of 290 patients surviving recent AMI, BTL measured on admission was a strong predictor of all-cause [hazard ratio (HR) [95% confidence interval (CI)]: 3.21 [1.46–7.06], *P* = 0.004] and cardiovascular mortality (HR [95% CI]: 3.96 [1.65–9.53], *P* = 0.002) 1 year after AMI (for comparisons of short vs. long BTL, as defined by a T/S ratio cut-off of 0.916, calculated using receiver operating characteristic analysis; *P* adjusted for age and other predictors). To explore the biological meaning of these findings, BTL was quantified in 727 consecutive patients undergoing coronary artery bypass grafting (CABG), and superoxide (O_2_^.-^) was measured in peripheral blood mononuclear cells (PBMNC). VTL/vascular O_2_^.-^ were quantified in saphenous vein (SV) and mammary artery (IMA) segments. Patients were genotyped for functional genetic polymorphisms in *P22^ph^°^x^* (activating NADPH-oxidases) and vascular smooth muscle cells (VSMC) selected by genotype were cultured from vascular tissue. Short BTL was associated with high O_2_^.-^ in PBMNC (*P* = 0.04) but not in vessels, whereas VTL was related to O_2_^.-^ in IMA (*ρ* = −0.49, *P* = 0.004) and SV (*ρ* = −0.52, *P* = 0.01). Angiotensin II (AngII) incubation of VSMC (30 days), as a means of stimulating NADPH-oxidases, increased O_2_^.-^ and reduced TL in carriers of the high-responsiveness *P22^ph^°^x^* alleles (*P* = 0.007).

**Conclusion:**

BTL predicts cardiovascular outcomes post-AMI, independently of age, whereas VTL is a tissue-specific (rather than a global) biomarker of vascular oxidative stress. The lack of a strong association between BTL and VTL reveals the importance of systemic vs. vascular factors in determining clinical outcomes after AMI.

## Introduction

Acute myocardial infarction (AMI) remains a major public health problem and while early reperfusion strategies have reduced in-hospital mortality to ∼5%, 1-year post AMI mortality is still ∼9%.^1^ Although risk factors including age, location and size of infarct, haemodynamic status, and renal disease predict mortality post-AMI^2^; stratifying patients with differential residual risk remains challenging. Novel predictive biomarkers that facilitate better risk stratification and personalized patient management are needed. Such markers could be particularly useful in targeting patient selection for upcoming therapies such as monoclonal antibodies against protein convertase subtilisin/kexin type 9 (PCSK9), which may significantly reduce cardiovascular mortality,^3^ but will also place great demands on healthcare expenditure.

The pathogenesis of AMI is characterized by monocyte/macrophage infiltration of the myocardium,^4^ while blood leukocyte and neutrophil counts during acute AMI are known to predict clinical outcome.^5^ In addition, endothelial dysfunction, as estimated by impaired flow-mediated dilatation (FMD) of the brachial artery, is linked to increased risk of cardiovascular events.^6^ Although oxidative stress plays an important role in myocardial reperfusion injury,^7^ existing plasma biomarkers of oxidative stress are typically oxidation products influenced by both their systemic production and elimination rate,^8^ and do not necessarily reflect vascular redox state.^8^ Similarly, while some systemic markers of oxidative stress (such as oxidized plasma aminothiols cystine and glutathione) predict clinical outcome in coronary artery disease (CAD),^9^ it is unclear whether their predictive value is due to their capacity to characterize systemic oxidative stress or other biological processes.

Telomeres are nucleoprotein structures at chromosomal ends, which serve to protect chromosomes from recombination or degradation, ensuring integrity during replication.^10^^,^^11^ After each replication cycle, telomere length (TL) is reduced; hence TL is considered to reflect cellular age.^11^ Although experimental models suggest that oxidative stress shortens TL *in vitro*,^12^ it is unclear whether TL can be used as a marker of oxidative stress *in vivo* or even as a predictive biomarker in conditions involving redox signalling, such as AMI. It is also unclear whether blood TL (BTL) is tissue-specific or whether it is a global biomarker of oxidative stress, thereby reflecting vascular redox state. Blood BTL is driven by TL of white blood cells (WBCs), and reportedly correlates with TL of aortic tissue,^13^ a correlation which may be influenced, however, by inflammatory cell infiltration of the aortic wall.

We explore the role of BTL as a biomarker of systemic and vascular oxidative stress in humans, and examine its predictive value in patients with AMI.

## Methods

### Patients

*Cohort 1*: We prospectively recruited 290 patients admitted with AMI [ST segment elevation AMI (STEMI, *n* = 163) or non-STEMI (NSTEMI, *n* = 127), *Table 1*]. All patients were approached for enrolment as soon as they were able to give informed consent after receiving appropriate revascularization [primary percutaneous coronary intervention (PCI) or thrombolysis]. AMI was diagnosed according to clinical guidelines.^14^ In particular, STEMI was diagnosed in patients with new ST segment elevation ≥2 mm in ≥2 contiguous precordial leads, or ≥1 mm in ≥2 continuous limb leads or when new left bundle branch block in the presence of compatible symptoms was found on the qualifying ECG. STEMI patients received either primary PCI (56.1%) or thrombolysis followed by rescue PCI within 24 h of their admission (43.9%). NSTEMI was diagnosed in patients who presented with chest pain with ECG abnormalities suggesting acute ischaemic heart disease (i.e. no persistent ST-segment elevation but rather persistent or transient ST-segment depression or T-wave inversion, flat T waves, pseudo-normalization of T waves, or non-specific ECG changes; or even normal ECG at presentation) and elevated plasma biomarkers of myocardial damage [Troponin I (TnI) with at least one determination greater than the upper limit of normal values as defined by the clinical laboratory]. Patients with ischaemic ECG abnormalities but no symptoms (silent ischaemia) were also included in this category, if blood TnI was greater than the upper limit of normal values as defined by the clinical laboratory. All NSTEMI patients received standard care and underwent PCI within 24 h after hospital admission. Principal exclusion criteria included active infection or chronic inflammatory disease, any significant systemic disease, hepatic or overt renal dysfunction (serum creatinine > 2.5mg/dL), malignancy, major surgery in the previous month or need for emergency CABG. Moreover, patients who presented with cardiogenic shock or died during the first 48 h of their hospital stay or during revascularization were also excluded from the study, as obtaining informed consent from these patients was not possible.

**Table 1:** Participant demographic characteristics

	Cohort 1	Cohort 2
Participants (*n*)	290	727
Age (years)	63.0 *±* 12.7	65.9 ± 9.6
Male gender (%)	85.2	80.9
Hypertension (%)	61.4	71.1
Hyperlipidaemia (%)	58.3	64.2
T2DM (%)	27.5	27.4
Active smoking (%)	62.4	20.5
BMI (kg/m^2^)	26.7 ± 2.3	28.2 ± 4.2
BTL^a^	1.08 [0.41–2.66]	0.42 [0.04–0.99]
Type of AMI		
STEMI (%)	56.2	—
NSTEMI (%)	43.8	—
Management of STEMI		
Primary PCI (%)	56.1	—
Thrombolysis and PCI within 24h(%)	43.9	—
Medication		
Aspirin (%)	53.2	77
Clopidogrel (%)	2.8	31.8
ACEi/ARBs (%)	57.2/9.4	50.7/14.0
Statins (%)	57.9	80.6
βblockers (%)	72.1	68.4
CCB (%)	19.3	27.6
Insulin (%)	2.8	5.7
Oral hypoglycaemic (%)	21.3	19.3

Values presented as mean ± standard deviation.

T2DM, type 2 diabetes mellitus; BMI, body mass index; AMI, acute myocardial infarction; STEMI, ST segment elevation myocardial infarction; NSTEMI, non-STEMI; PCI, percutaneous coronary intervention; ACEi, angiotensin converting enzyme inhibitors; ARB, angiotensin receptor blockers; CCB, calcium channel blockers; BTL, blood telomere length.

a Median [10th–90th percentile].

Follow-up data were obtained during outpatient consultation or telephone interview with the patient or, in the case of death, from hospital records and consultation with family members and/or attending physicians. All-cause mortality, cardiovascular mortality, and non-fatal AMI during the first year of follow-up were used as endpoints.

*Cohort 2:* This included 727 patients undergoing elective CABG surgery at Oxford University Hospitals NHS Foundation Trust. Patients were prospectively recruited and exclusion criteria included inflammatory, neoplastic, renal, or hepatic disease. During surgery, saphenous vein (SV) and internal mammary artery (IMA) segments were obtained and processed immediately after harvest for O_2_^.-^ measurements or snap-frozen for DNA extraction. Vascular smooth muscle cells (VSMC) were isolated routinely from SV samples and used for mechanistic experiments based on their genotype. The demographic characteristics of the participants are presented in *Table 1*.

Study protocols were approved by the respective institutional ethics committees and the patients gave written informed consent prior to their enrolment. The studies were in accordance with the Declaration of Helsinki.

### Blood sampling and circulating biomarker measurements

For Cohort 1, blood samples for measurement of creatinine, brain natriuretic peptide (BNP) and C-reactive protein with a high sensitivity assay (hsCRP) were collected on hospital admission as part of routine clinical care, but before any in-hospital treatment was initiated. Daily blood samples for plasma biomarker measurements were collected throughout the patients’ hospital stay; blood samples for DNA extraction and BTL quantification were also collected. For Cohort 2, blood samples were collected prior to cardiac surgery for plasma biomarker measurements and DNA extraction, as well as for isolation of peripheral blood mononuclear cells (PBMNC) (see Supplementary material online, *Methods*).

### Isolation of peripheral blood mononuclear cells

PBMNC were isolated from blood samples of 128 patients in Cohort 2 (see Supplementary material online, *Table S2*) using density gradient centrifugation (see Supplementary material online, *Methods*).

### DNA extraction and genotyping

DNA was extracted using commercially available kits and genotyping was performed using TaqMan probes (see Supplementary material online, *Methods*).

Measurement of TL: TL was measured by quantitative PCR as described by Cawthon *et al* (see Supplementary material online, *Methods*).^15^ BTL was measured in all patients in Cohorts 1 and 2, while VTL was quantified in the IMA of 32 and in the SV of 24 patients from Cohort 2 (see Supplementary material online, *Table S2*).

### Assessment of endothelial function

Vascular endothelial function was evaluated non-invasively by measuring the FMD of the brachial artery as previously described^16^^,^^17^ (see Supplementary material online, *Methods*).

### Harvesting and processing of human vessels

IMA and SV samples were harvested during CABG and processed as previously described (see Supplementary material online).^18^

### Superoxide measurements in human vessels

Vascular O_2_^.-^ production was measured in fresh, intact vascular segments by lucigenin (Sigma, 5 μmol/L)-enhanced chemiluminescence, as previously described,^19^ and the activity of NADPH-oxidases was evaluated by measuring the NADPH-stimulated O_2_^.-^ (see Supplementary material online, *Method*).

### Quantification of DNA oxidative damage

DNA damage was evaluated by using the comet assay (CometAssay kit,Trevigen, Maryland, USA) (see Supplementary material online, *Methods*).

### Telomere restriction fragment analysis

Telomere length was measured using Telo TAGGG Telomere Length Assay kit (Sigma), according to the instructions provided by the manufacturer. Total telomere length, expressed as kpb, was finally quantified using TeloTool software (see Supplementary material online, *Methods*).

### NADPH-oxidases activity in peripheral blood mononuclear cells

O_2_^.-^ production in PBMNC was also measured using a lucigenin-enhanced chemiluminescence assay^20^ (see Supplementary material online, *Methods*).

### Cell culture experiments

VSMC from SV specimens of six patients in Cohort 2 (see Supplementary material online, *Table S2*), selected on the basis of *CYBA* genotypes to be homozygotes for either rs4673**T**/rs1049255**A** or rs4673**C**/rs1049255**G** (see Supplementary material online, *Isolation Method*), were used to examine the relationship between oxidative stress and TL. VSMC were cultured with/without angiotensin II (AngII) 100 nM (Sigma) for 30 days, alternating on 24 h cycles to stimulate NADPH-oxidases, in order to examine the effect of NADPH-oxidases–derived O_2_^.-^ on TL.^21^ At the end of the experiment, DNA was isolated and TL was assessed (see Supplementary material online).

*Superoxide measurement in VSMC*: O_2_^.-^ production in VSMCs was measured using lucigenin-enhanced chemiluminescence (see Supplementary material online, *Methods*).

### Antioxidant measurements

Plasma total antioxidant capacity (TAC) and superoxide dismutase (SOD) activity, as well as vascular SOD activity, were measured by using commercially available assays (see Supplementary material online).

### Statistical analysis

Continuous variables were tested for normality using the Kolmogorov–Smirnov test. Non-normally distributed variables are presented as median [25th–75th percentile] or on a log-transformed scale. Normally distributed variables are presented as mean ± standard deviation (SD) or mean [95% confidence interval (CI)] as stated.

Comparisons of normally distributed variables between groups were performed by using unpaired *t*-tests or one-way analysis of variance. Comparisons of non-normally distributed variables between groups were performed by using the Mann–Whitney *U* test or the Kruskal–Wallis test as appropriate.

Power calculations for Cohort 1 showed that with 290 patients, there was a statistical power of 0.82 to detect a 42.5% difference in the survival rates between patients with long vs. short BTL [35% belonging in the short BTL group as defined by receiver operating characteristic (ROC) analysis], with α = 0.05. For Cohort 2, we estimated that 199 patients with short and 370 patients with long BTL would allow detection of 8.3% difference in the NADPH-stimulated O_2_^.-^ between patients with long vs. short BTL, assuming a SD of 65, with power 90% and α = 0.05. For the subset of Cohort 2 for which data on VTL was available, with *n* = 29 patients there was a statistical power of 0.80 to detect a significant correlation between VTL and NADPH-stimulated O_2_^.-^ generation with *r* = 0.50 and α = 0.05. For the *ex vivo* VMSC experiments, power calculations showed that with *n* = 4 pairs we could detect a 38% change in T/S ratio after exposure to AngII, with a power of 0.90 and *a* = 0.05.

Pearson’s r or Spearman’s *ρ* simple correlation coefficients were estimated, as appropriate. To evaluate the association between BTL and clinical outcome, ROC analysis was performed and an optimal cut-off BTL value of 0.916 was selected. The study population was then divided into 2 groups with long (T/S ratio ≥ 0.916) or short (T/S ratio < 0.916) BTL. Comparisons between Kaplan–Meier curves defined as above or below the BTL cut-off were performed originally using the Breslow test (given that more deaths were expected in the early post-AMI period). The predictive value of BTL on survival was then tested using cox-regression; patient demographic characteristics were included as covariates (e.g. age, gender, hypertension, diabetes mellitus, dyslipidaemia, smoking, body mass index, medication), as well as other known predictors of outcome post-AMI (e.g. peak troponin levels, hsCRP, creatinine and BNP) that showed an association (*P* < 0.10 in univariate Cox models) with clinical outcome (mortality and non-fatal AMI). To assess the incremental prognostic value of BTL beyond established risk factors, we compared the ROC curves (established predictors vs. established predictors plus BTL) using C-statistic. All statistical analyses were performed by using SPSS version 22.0 (SPSS, Chicago, IL, USA) or Stata version 14.0 (Stata Corp Inc., College Station, TX, USA, used for c-statistics) and the tests were two-tailed. *P*-values < 0.05 were considered significant.

## Results

**Figure 1:**
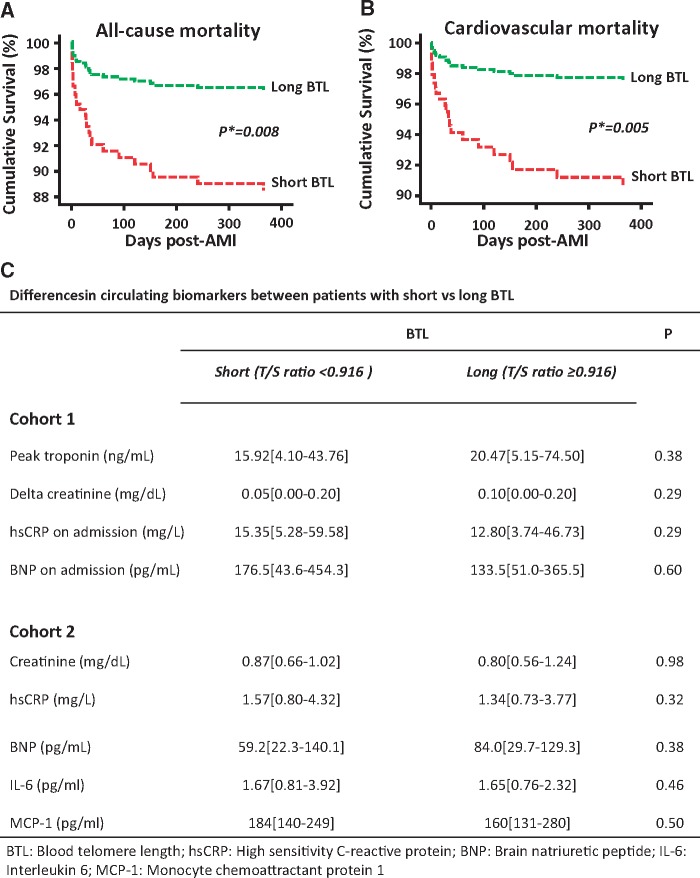
In Cohort 1, short blood telomere length (BTL, T/S ratio < 0.916) was predictive of all-cause (*A*) and cardiovascular mortality (*B*) during the first year after acute myocardial infarction (AMI). BTL in Cohort 1 was not associated with known predictors of outcome following AMI, and these analyses were replicated in Cohort 2 to account for potential differences between stable and unstable coronary artery disease (CAD), as described in Panel *C* (data presented as median [25th–75th percentile]). Delta-creatinine: change in creatinine from admission to the peak value during hospital stay; hsCRP, high sensitivity C-reactive protein; BNP, brain natriuretic peptide; IL-6, interleukin 6; MCP-1, monocyte chemoattractant protein 1; *P-values in Kaplan–Meier curves shown in Panels *A* and *B* derived from Breslow test; *P*-values in Panel *C* derived from Mann–Whitney *U* tests.

The demographics of the study participants are presented in *Table 1*. In Cohort 1, during the 1-year follow-up period there were 30 deaths (12.3%), 26 of which were due to cardiovascular causes. There were also 20 new non-fatal AMIs and 22 new non-fatal acute coronary syndromes (defined by symptoms of chest pain, ECG changes indicative of ischaemia and negative cardiac biomarkers). The unadjusted predictors of all-cause and cardiovascular mortality are presented in *Table 2*. To explore the predictive value of BTL post-AMI, the study population was split in tertiles of BTL and a survival analysis was performed (see Supplementary material online, *Figure S1*). Patients in the short BTL tertile had a significantly higher risk of both all-cause and cardiovascular mortality compared to those in the mid ± long BTL tertiles (*P* = 0.02 for both endpoints), while no significant difference was observed between the mid vs. long BTL tertiles (*P* = 0.86 for all-cause and 0.45 for cardiovascular mortality), suggesting the presence of a biological threshold effect. Next, ROC analysis was performed to identify a BTL cut-off value with adequate sensitivity and specificity for cardiovascular and all-cause mortality (see Supplementary material online, *Figure S1*). We found that a BTL cut-off value of 0.916 would have 66.7% sensitivity and 59.7% specificity to predict all-cause mortality [area under the curve (AUC) = 0.62, *P* = 0.03], whereas the same cut-off value would lead to 69.2% sensitivity and 59.6% specificity for prediction of cardiovascular mortality (AUC = 0.64, *P* = 0.02, see Supplementary material online, *Figure S1*). By using the telomere restriction fragment assay, the absolute telomere length for this cut-off was estimated to be 9.29kb (see Supplementary material online, *Figure S1*). Despite the weak AUC for BTL as a sole predictor of all-cause or cardiac mortality, we then showed that the AUC for an optimum model predicting all-cause mortality using all available demographic characteristics, relevant biochemical variables and medication, was AUC [95% CI]: 0.86 [0.79–0.94], and there was a small but significant improvement of the model to 0.89 [0.84–0.95], and delta[AUC] = 0.03, *P* = 0.048. For cardiovascular mortality, the AUC for the same risk factors model was 0.88 [0.80–0.96], and that was also improved to 0.92 [0.87–0.98], with delta (AUC) = 0.04, *P* = 0.04 (see Supplementary material online, *Figure S1*). The population was then divided into 2 patient groups [those with short BTL (T/S ratio < 0.916) and those with long BTL (T/S ratio ≥ 0.916)], and the analysis of Kaplan–Meier curves revealed significantly greater all-cause and cardiovascular mortality in those patients with short BTL (*Figure 1A and B*). Demographic characteristics were similar in the two patient groups (see Supplementary material online, *Table S1*). To examine whether the observed association between BTL and clinical outcome was driven by indirect associations of BTL with known predictors of survival post-AMI, we searched for correlations between BTL and other known outcome predictors. We found no association between BTL and TnI, creatinine, hsCRP, and BNP (*Figure 1C*) and no difference in BTL between STEMI and NSTEMI patients (*P* = 0.49). In addition, treatment of AMI (i.e. either PCI or thrombolysis) was not a significant predictor of either cardiovascular (HR [95% CI]: 1.41 [0.66–3.02], *P* = 0.37) or all-cause (HR [95% CI]: 1.33 [0.67–2.64], *P* = 0.39) mortality in univariate Cox analysis. To exclude the possibility that the lack of such associations was due to the rapidly changing circulating biomarkers during AMI, we confirmed these findings in Cohort 2, comprising patients with stable CAD undergoing elective CABG. In these patients, BTL was not significantly associated with either plasma creatinine or the inflammatory markers IL6 and MCP1 (Cohort 2, *Figure 1C*). BTL was not related to non-fatal AMI in univariate analysis (HR [95% CI]: 2.33 [0.47–11.53], *P* = 0.30). Interestingly, there was no significant association between BTL and chronological age in either Cohort 1 (*ρ* = 0.05, *P* = 0.41) or Cohort 2 (*ρ* = −0.04, *P* = 0.33), possibly due to the narrow age range of the 2 cohorts (median [25th−75th percentile]: 62 [53–73] years for Cohort 1 and 67 [59–73] years for Cohort 2). Of note, no significant interaction was found between age and BTL with regards to prediction of all-cause or cardiovascular mortality (*P* = 0.78 and *P* = 0.50 for age x BTL interaction, respectively). Additionally, concomitant mediation such as angiotensin converting enzyme inhibitors (ACEi) and angiotensin receptor blockers (ARB) did not influence BTL (see Supplementary material online, *Figure S3*).

**Table 2:** Predictive value of blood telomere length post-acute myocardial infarction (Cohort 1)

	Predictors	HR [95% CI], P*
All-cause mortality	Univariate analysis	
	BTL^a^	2.73 [1.28–5.83], *P* = 0.01
	Age (per decade)	1.98 [1.40–2.81], *P* < 0.001
	BNP on admission (per 100 pg/mL)	1.07 [1.05–1.09], *P* < 0.001
	Change in creatinine (per mg/dL)	1.78 [1.45–2.17], *P* < 0.001
	Peak troponin (per 10 ng/mL)	1.02 [1.01–1.04], *P* = 0.03
	Type of AMI (STEMI vs. NSTEMI)	0.50 [0.24–1.03], *P* = 0.06
	Number of diseased coronaries	1.36 [1.01–1.83], *P* = 0.04
	hsCRP on admission (per 10 mg/L)	1.06 [1.01–1.11], *P* = 0.02
	Multivariable analysis	
	BTL^a^	3.21 [1.46–7.06], *P* = 0.004
	Age (per decade)	1.85 [1.25–2.73], *P* = 0.002
	BNP on admission (per 100 pg/mL)	1.05 [1.02–1.08], *P* = 0.001
	Change in creatinine (per mg/dL)	1.48 [1.14–1.94], *P* = 0.003
	Peak troponin (per 10 ng/mL***)***	1.03 [1.00–1.06], *P* = 0.07
	Type of AMI (STEMI vs. NSTEMI)	1.35 [0.55–3.31], *P* = 0.51
	Number of diseased coronaries	0.183 [0.54–1.25], *P* = 0.37
	hsCRP on admission (per 10 mg/L)	1.03 [0.97–1.10], *P* = 0.31
Cardiovascular mortality	Univariate analysis	
	BTL^a^	3.08 [1.34–7.09], *P* = 0.008
	Age (per decade)	2.17 [1.47–3.20], *P*<0.001
	BNP on admission (per 100 pg/mL)	1.07 [1.05–1.09], *P*<0.001
	Change in creatinine (per mg/dL)	1.85 [1.50–2.27], *P*<0.001
	Peak troponin (per 10 ng/mL)	1.03 [1.01–1.05], *P* = 0.01
	Type of AMI (STEMI vs. NSTEMI)	0.47 [0.21–1.03], *P* = 0.06
	Number of diseased coronaries	0.38 [0.12–1.16], *P* = 0.09
	hsCRP on admission (per 10 mg/L)	1.05 [1.00–1.11], *P* = 0.07
	Multivariable analysis	
	BTL^a^	3.96 [1.65–9.53], *P* = 0.002
	Age (per decade)	2.08 [1.34–3.22], *P* = 0.001
	BNP on admission (per100 pg/mL)	1.06 [1.03–1.09], *P* < 0.001
	Change in creatinine (per mg/dL)	1.55 [1.18–2.05], *P* = 0.002
	Peak troponin (per 10 ng/mL)	1.04 [1.01–1.07], *P* = 0.02
	Type of AMI (STEMI vs. NSTEMI)	1.44 [0.54–3.88], *P* = 0.46
	Number of diseased coronaries	0.70 [0.45–1.10], *P* = 0.13
	hsCRP on admission (per 10 mg/L)	1.02 [0.95–1.09], *P* = 0.66

BNP, brain natriuretic peptide; AMI, acute myocardial infarction; hsCRP, high sensitivity C reactive protein; HR, hazard ratio; CI, confidence interval; STEMI, ST segment elevation myocardial infarction; NSTEMI, non-STEMI.

a Blood telomere length, comparisons of long BTL (T/S ratio ≥ 0.916) vs. short BTL (T/S ratio < 0.916)

* *P* values by cox-regression.

**Figure 2:**
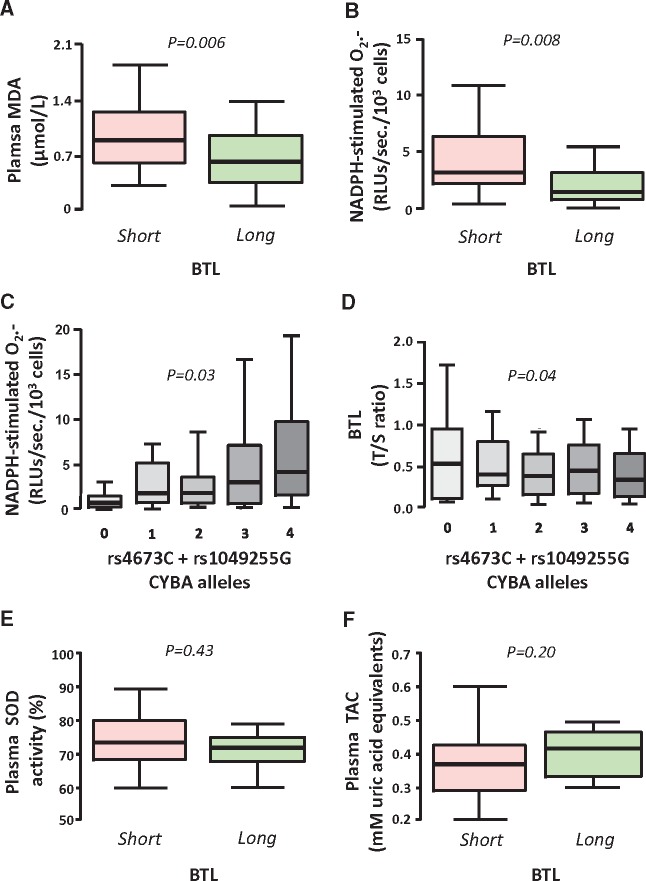
In Cohort 2, short blood telomere length (BTL < 0.916) was associated with elevated plasma malondialdehyde (MDA, *A*) and increased NADPH-stimulated superoxide (O_2_^.-^) production by peripheral blood mononuclear cells (PBMNC, *B*) in patients undergoing coronary artery bypass grafting surgery. The combined effect of two functional single nucleotide polymorphisms of the *CYBA* gene encoding the p22^ph^°^x^ subunit of NADPH-oxidases (rs4673 and rs1049255) affected O_2_^.-^ production in PBMNC, in a way that the number of rs4673C and rs1049255G alleles would be positively related with NADPH-stimulated O_2_^.-^ in these cells (*C*). Mendelian randomization approach revealed that this additive genetic effect resulted in reduced BTL (*D*). BTL was not associated with either plasma superoxide dismutase (SOD) activity (*E*) or plasma total antioxidant capacity (TAC, *F*). Values are presented as median [25th–75th percentile]; *P*-values are calculated using Mann–Whitney *U* tests for Panels *A*, *B*, and *E*–*F* or Kruskal–Wallis tests for Panels *C* and *D*.

In order to understand the biological meaning of BTL, we next focused on Cohort 2, using clinical tissue samples and bioassays to address the hypothesis that BTL is a biomarker of systemic oxidative stress. There was a significant, though weak, inverse association between BTL and malondialdehyde (MDA), an established systemic biomarker of lipid peroxidation (*Figure 2A*). However, since BTL principally reflects TL in circulating WBCs, we next examined the association of BTL with O_2_^.-^ generation from PBMNC isolated from 128 patients in Cohort 2, and observed a striking association between BTL and PBMNC O_2_^.-^ generation (*Figure 2B*). As associations do not necessarily reflect causality, we explored the effect of two functional SNPs in the *CYBA* gene (rs4673/rs1049255) on BTL. The additive model of these two SNPs strongly affected O_2_^.-^ generation from NADPH oxidases in PBMNC (*Figure 2C*) and BTL (*Figure 2D*). In order to explore the role of systemic antioxidant defences in the regulation of BTL, we next quantified both plasma TAC and SOD activity, and found no significant correlation with BTL, indicating that it is the net bioavailability of O_2_^.-^ that determines BTL, rather than individual components of the endogenous antioxidant defences (*Figure 2E and F*).

**Figure 3:**
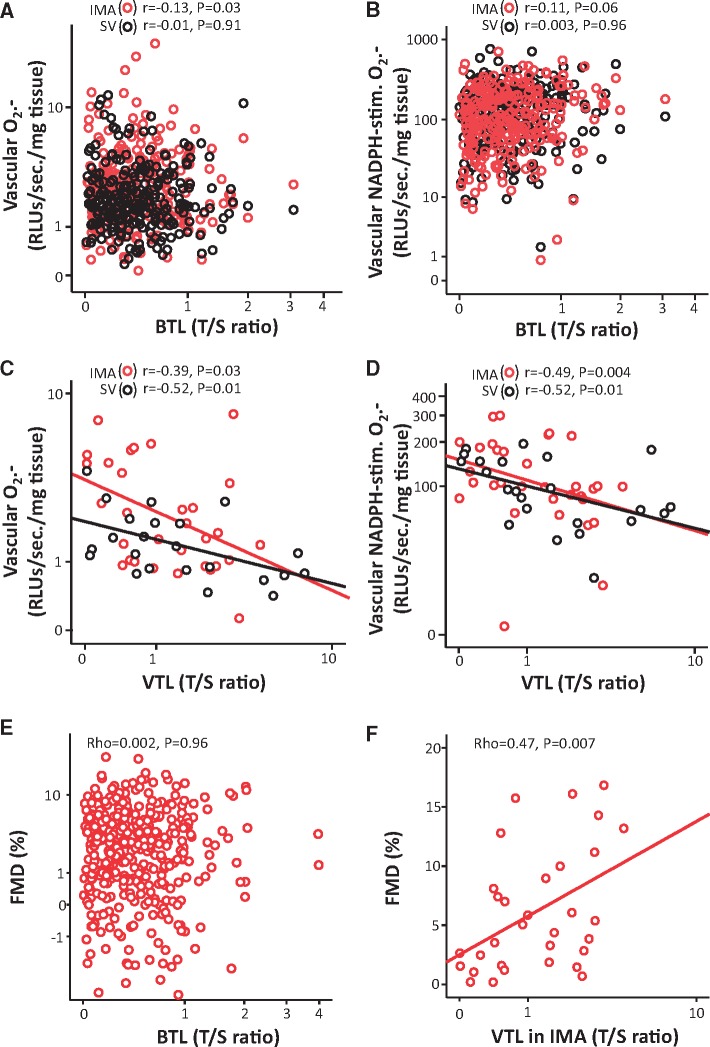
In the *ex vivo* arm of Cohort 2, blood telomere length (BTL) was not associated with basal or NADPH-stimulated superoxide (O_2_^.-^) in saphenous vein (SV) or internal mammary artery (IMA) segments (*A* and *B*). On the contrary, there was an inverse association between vascular telomere length (VTL) and both basal (*C*) and NADPH-stimulated O_2_^.-^ (*D*) in the respective vascular tissue, in a subgroup of 32 IMA & 24 SV. Shortened VTL in IMA was associated with impaired flow mediated dilatation (FMD) of the brachial artery *in vivo* (*F*), in contrast with BTL (*E*).

We also examined whether BTL could reflect vascular redox state. However, BTL was not related to vascular redox state, quantified in vessels from patients in Cohort 2 (*Figure 3A* and *B*). We also quantified TL in IMA from 32 patients and SV from 24 patients of Cohort 2, and observed a striking inverse association between VTL and basal or NADPH-stimulated O_2_^.-^ generation within both tissues (*Figure 3C* and *D*). Interestingly, BTL was not related with TL in SVs (*ρ* = 0.24, *P* = 0.37) or IMAs (*ρ* = 0.39, *P* = 0.14), suggesting that the association of TL with oxidative stress is tissue-specific. Similarly, BTL was not related to endothelial function as evaluated by FMD of the brachial artery (*Figure 3E*), contrary to arterial TL (measured in IMAs) (*Figure 3F*), confirming that TL reflects redox state in a tissue-specific way. Quantification of vascular SOD activity revealed no significant association with VTL in either SV or IMA (see Supplementary material online, *Figure S2*), confirming that the overall bioavailability of O_2_^.-^, rather than individual antioxidant enzymes, determines VTL.

In order to evaluate potential patient characteristics that may affect the interaction of BTL with VTL, we examined whether atherosclerotic plaque burden or systemic inflammation may influence the correlation between BTL and VTL. We found that the angiographic extent of coronary atherosclerosis (classified as 1-, 2-, or 3-vessel disease) was not related with any TL measurements (*ρ* = −0.11, *P* = 0.60 for VTL in SV, *ρ* = 0.08, *P* = 0.70 for VTL in IMA, *ρ* = 0.04, *P* = 0.35 for BTL). Additionally, WBC count was not related with VTL in IMA (*ρ* = −0.20, *P* = 0.58), VTL in SV (*ρ* = −0.27, *P* = 0.37) or BTL (*ρ* = −0.07, *P* = 0.14). Similarly, we also found that hsCRP was not related to VTL in IMA (*ρ* = −0.25, *P* = 0.29), SV (*ρ* = 0.08, *P* = 0.72) or BTL (*ρ* = −0.06, *P* = 0.13). These findings suggest that neither coronary atherosclerosis burden nor systemic inflammation influence the interactions between VTL and BTL in our study. Furthermore, ACEi/ARB treatment was not related to BTL in either Cohort 1 or Cohort 2 (see Supplementary material online, *Figure S3*), or VTL in IMA & SV (see Supplementary material online, *Figure S4*).

**Figure 4:**
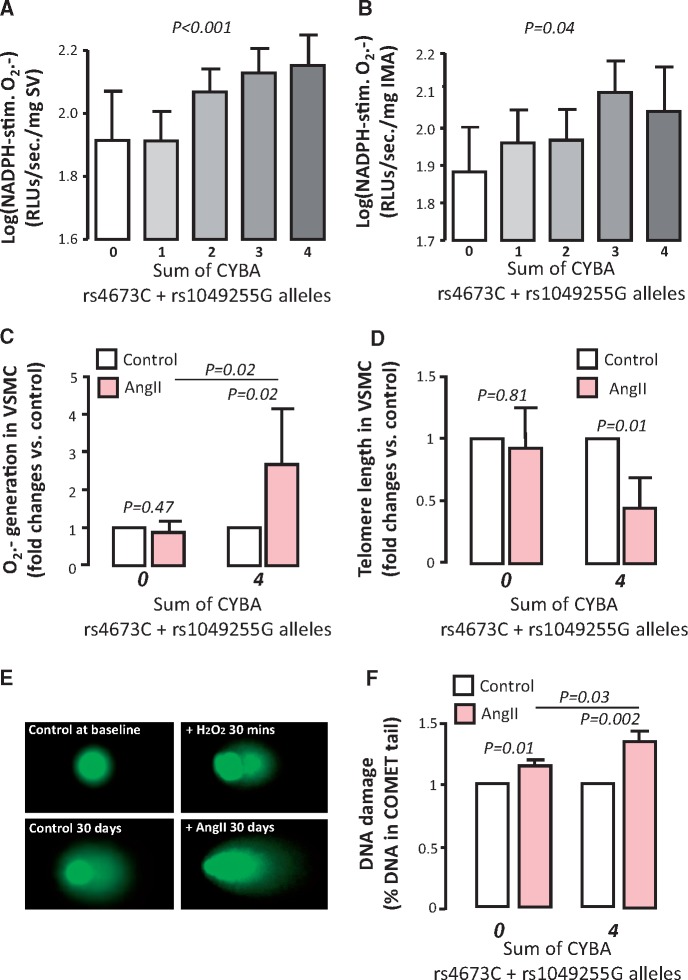
The combined effect of the two functional single nucleotide polymorphisms located in the *CYBA* gene encoding the p22^ph^°^x^ subunit of NADPH oxidase (rs4673 and rs1049255) affected superoxide (O_2_^.-^) production in both saphenous veins (SV, *A*) and internal mammary arteries (IMA, *B*) in Cohort 2, in a way that the number of rs4673C and rs1049255G alleles were positively related with NADPH-stimulated O_2_^.-^ in these vessels. Following a Mendelian randomization approach using these two SNPs, vascular smooth muscle cells (VSMC) isolated from homozygote patients for rs4673**T** and rs1049255**A** (low vascular NADPH-oxidases activity, 0 risk alleles) vs. VSMC from homozygote patients for rs4673**C** and rs1049255**G** (high vascular NADPH-oxidases activity, 4 risk alleles) were retrieved. Following 24 h stimulation of primary VSMC from the two extreme *CYBA* genotypes with Angiotensin II (AngII) 100 nM, only cells with the 4 risk alleles increased their O_2_^.-^ generation compared to non-stimulated controls, while those with 0 risk alleles were not influenced by AngII stimulation (*C*, *n* = 8 pairs for each genotype). Accordingly, telomere length (TL) was significantly shortened following chronic (30 days on a 24 h on/off cycle) AngII 100 nM stimulation only in VSMC with 4 risk alleles (*D*, *n* = 8 pairs for each genotype). Using the Comet assay, we observed that AngII incubation significantly increased DNA damage in VSMC, an effect that was significantly greater in cells with 4 *CYBA* risk alleles (rs4673C and rs1049255G) vs. those with 0 CYBA alleles (*E* and *F*, *n* = 10 pairs per genotype). As positive control, we used VSMC exposed to H_2_O_2_ 100 μM for 30 min, and examples of images are presented in Panel *E*. *P*-values in Panels *A* and *B* are calculated using Kruskal–Wallis test, and presented as logarithmic values. *P*-values in Panels *C*, *D* and *F* are calculated using Wilcoxon signed rank test and presented as fold changes versus control or risk alleles post treatment. Values presented as mean (95% confidence interval).

To further confirm that oxidative stress drives TL, we selected VSMC that were isolated from SV segments of patients with extreme *CYBA* genotypes (rs4673T/rs1049255A) vs. (rs4673C/rs1049255G) which are known to significantly affect NADPH-oxidases activity in human arteries and veins (*Figure 4A* and *B*). O_2_^.-^ production in cells from homozygote patients for both rs4673T and rs1049255A (0 high O_2_^.-^ alleles) was unaffected after AngII stimulation (100 nM), while homozygotes for rs4673C and rs1049255G (4 high O_2_^.-^ alleles) had significantly increased O_2_^.−^ generation in response to AngII (*Figure 4C*). To show that O_2_^.-^ can actually modify TL in these cells, we then performed a long-term experiment in which we exposed VSMC from patients in Cohort 2, based on their *CYBA* genotype, to AngII 100nM (or not) for 30 days on a 24 h on/off cycle. At the end of the incubation period, VSMC from patients with 0 alleles had no change in TL compared to controls, while cells from patients with 4 alleles showed a significant reduction of TL (*Figure 4D*).

In a separate experiment, to confirm that 30 days incubation of VSMCs from these patients with AngII 100nM (on a 24 h on/off cycle), leads to significant DNA damage, we employed the Comet assay to assess DNA damage. We observed that AngII leads to significant DNA damage in cells from both genotypes (0 or 4 CYBA risk alleles), but the effect in cells with 4 CYBA risk alleles was significantly greater compared to those with 0 alleles (in 10 independent experiments from each genotype), confirming the link between oxidative stress (mainly O_2_^.-^ but probably also other reactive oxygen species) and DNA damage in this model (*Figure 4E* and *F*). Taken together, our findings confirm that TL is determined by the cumulative effect of oxidative stress over time, in a tissue-specific way, and it can be used as a new biomarker of oxidative stress in humans, at least for the specific tissue type where it is measured.

## Discussion

**Figure 5:**
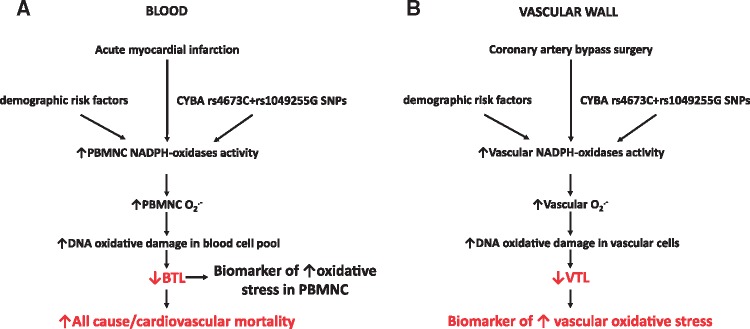
Summary of the proposed mechanism. Blood telomere length (BTL) is a biomarker of superoxide (O_2_^.-^) production by peripheral blood mononuclear cells (PBMNC). In these cells, production of O_2_^.-^ is determined by NADPH-oxidases activity, which is, in turn, influenced by demographic and genetic parameters such as the CYBA single nucleotide polymorphisms (SNPs) rs4673 and rs1049255 encoding for the p22^ph^°^x^ subunit of NADPH-oxidases. Importantly, acute myocardial infarction may stimulate oxidative activation of PBMNC; this is accompanied by shortening of BTL, which serves as a predictor of all-cause and cardiovascular mortality following myocardial infarction (*A*). Accordingly, demographic and genetic factors co-influence NADPH-oxidases activity in the vascular wall of humans undergoing coronary artery bypass surgery, which then inflicts DNA oxidative damage resulting in shortening of vascular telomere length (VTL). Therefore, VTL comprises a biomarker of increased vascular oxidative stress, in a tissue-specific manner (*B*).

In this study, we investigated the value of BTL as a biomarker of systemic oxidative stress and explored its predictive value in patients post-AMI. Short BTL was an independent predictor of all-cause and cardiovascular mortality, even after adjustment for demographics and well-known predictors of outcome such as renal function, troponin-I, BNP and age. BTL was only weakly associated with circulating MDA (a biomarker of lipid peroxidation), but it was strongly related with O_2_^.-^ production by PBMNC. VTL (but not BTL) was inversely correlated with vascular O_2_^.-^ production. The additive effect of functional SNPs in the *CYBA* locus encoding for the p22^ph^°^x^ subunit of NADPH-oxidases was associated with increased O_2_^.-^ in PBMNC and shortened BTL. Furthermore, AngII stimulation of primary VSMC at the high extreme of genetically pre-defined NADPH-oxidases activation resulted in higher O_2_^.-^ production and shorter TL compared to VSMC at the lowest extreme. In summary, we identify TL as a biomarker of tissue-specific oxidative stress and BTL specifically as a valuable predictor of mortality post-AMI (*Figure 5*).

Factors such as BNP, renal failure, peak troponin levels, and age have predictive value post-AMI.^22^ However, the high number of recurrent cardiovascular events following AMI highlights the need for additional biomarkers to appropriately risk stratify this patient population.^23^ Shortened TL is known to be associated with degenerative diseases, including cardiovascular disease such as advanced atherosclerosis.^24^ Conversely, although short TL may be associated with premature myocardial infarction,^25^ it is unclear whether BTL has any predictive role following AMI.

In our study, we firstly explored the predictive value of BTL over a 1-year period following AMI. BTL was not associated with known risk factors, but it was identified as an independent predictor of all-cause and cardiovascular mortality, adding significant predictive value beyond the known risk factors and routine biochemical biomarkers used in clinical practice for risk stratification. Interestingly, survival analysis revealed a threshold for the predictive value of BTL on survival post-AMI, below which there is a sharp increase in the mortality risk in these patients. The existence of this threshold supports the use of BTL as a dichotomous risk factor, even though the specific cut-off value proposed in this study might be specific to this study population, and will need validation in other cohorts.

According to current clinical guidelines,^26^ all patients should receive aggressive medical treatment, including appropriate revascularisation, following AMI. However, the significant residual risk highlights the need for novel biomarkers such as BTL to better characterize that risk. These additional biomarkers may also be useful in facilitating targeted prescription of novel, but costly therapies such as monoclonal antibodies against PCSK9, in appropriate patients.

Although BTL has previously been associated with cardiovascular risk, its role as a biomarker is unclear. In particular, it is unknown how BTL, an indicator predominantly of circulating blood cell biology, could mechanistically reflect vascular pathogenetic processes. In our study, we did not document a significant correlation between BTL and VTL in IMA or SV segments. BTL has been associated with VTL in the aortic wall,^13^ but that study was performed using aortic tissue from patients with abdominal aortic aneurysms as well as post-mortem aortic samples of ‘healthy’ individuals. The aortic wall often develops atherosclerosis, in contrast to SV and IMA, whereas stimuli such as hypertension increase aortic inflammatory infiltration even in individuals without atherosclerosis or other inflammatory vascular disease.^27^ Therefore, it is highly likely that the association between aortic VTL and BTL is driven by the TL of inflammatory cells that exist in the circulation and subsequently infiltrate the aorta, and it may not reflect the TL of vascular cells (endothelial cells, VSMC). Furthermore, the non-significant association between BTL and VTL in IMA and SV in our study was independent of coronary atherosclerosis burden and systemic inflammation (assessed by WBC count and circulating hsCRP levels). This further supports the notion that any observed correlation between VTL and BTL may be driven by vascular inflammation, whereas VTL of vessels free of inflammatory disease, such as IMA and SV, in not related with BTL, but rather influenced by local factors.

Reactive oxygen species induce direct damage and increase telomere sensitivity to nucleases, which combined with the inherent sensitivity of telomeres due to the end replication problem cause marked TL shortening *in vitro*.^28^ Therefore, we hypothesized that BTL reflects ‘systemic’ oxidative stress and it could be used as a biomarker of oxidative stress in the human vascular wall. Indeed, BTL was inversely associated with plasma MDA (a well-validated biomarker of systemic oxidative stress) as well as with increased O_2_^.-^ generation in PBMNC. On the contrary, the non-significant associations between BTL and plasma total antioxidant capacity or plasma SOD activity suggest that BTL is actually driven by the overall amount of O_2_^.-^ that results from the balance between endogenous pro-oxidant and anti-oxidant systems. As such, isolated enzymatic systems such as SOD are not sufficient to describe BTL, whereas MDA and PBMNC O_2_^.-^ generation are broader markers of oxidative stress and more likely to be linked to BTL.

To confirm that systemic oxidative stress is a causal determinant of BTL *in vivo*, we genotyped Cohort 2 for two functional SNPs of the *CYBA* locus (rs4673C, rs1049255G) encoding for p22^ph^°^x^ (a component of NOX1, NOX2, or NOX4 isoforms of NADPH oxidases),^29^ resulting in enhanced enzyme activity.^30^ Using such a model of Mendelian randomization, we identified causality behind our observed associations, given that NOX2 is the major NADPH-oxidase isoform in leucocytes and the major isoform activated in cardiovascular disease.^31^ The additive effect of the two SNPs resulted in increased O_2_^.-^ production from PBMNC as well as vascular segments. Increased O_2_^.-^ in PBMNC was accompanied by reduced BTL; therefore BTL could serve as a new biomarker of redox-sensitive PBMNC activation. Recently, systemic biomarkers of oxidative stress such as oxidised aminothiols have been shown to have predictive value in coronary artery disease.^9^ However, the origin of these biomarkers remains unclear and it is unknown what biological processes they describe. Since oxidative stress triggers monocyte activation and migration to the vascular wall, an effect crucial to plaque formation and rupture,^32^ the potential of BTL as a surrogate of oxidative activation of PBMNC may explain its predictive value post-AMI.

To further explore the notion that TL tracks oxidative stress *in vivo*, we focused on the relationship between TL and oxidative stress in vascular tissue. Reduced TL in endothelial cells and VSMC is associated with endothelial dysfunction, inflammation, cellular senescence and DNA damage resulting from oxidative stress *in vitro*.^12^^,^^33^ We consistently demonstrate that VTL in IMA and SV is inversely related with O_2_^.-^ generation in the respective tissues *ex vivo*. Additionally, reduced TL in IMA (but not BTL) was negatively correlated with endothelial function evaluated by FMD of the branchial artery. The lack of correlation between vascular SOD activity and VTL confirms the notion that VTL is determined by the tissue-specific net generation of reactive oxygen species, implying that individual anti-oxidant mechanisms do not significantly influence TL.

To confirm that the causal effect of oxidative stress on TL is indeed reproducible in the human vasculature, we incubated primary VSMC from patients carrying the two extreme *CYBA* genotypes with AngII (to stimulate NADPH-oxidases) for 30 days, and quantified TL changes. VSMC carrying the high O_2_^.-^ genotype responded to AngII stimulation by increasing O_2_^.−^ and reducing TL whereas cells carrying the low O_2_^.-^ genotype remained unaffected by AngII stimulation.

In our cell culture experiments, a detectable shortening of TL in VSMC was observed following treatment with AngII for a total of 30 days. However, as TL shortening is a cumulative process *in vivo*, reflecting the exposure of the tissue DNA to prolonged oxidative stress,^34^ it is unlikely that considerable TL shortening could be observed during the post-AMI period.

A limitation of the present study is the lack of a validation cohort for the mortality data, therefore extrapolation of the BTL cut-off value to other cohorts should be done with caution. Nevertheless, this does not limit the value of the finding that short BTL can be used as a marker of tissue-specific chronic oxidative stress with significant predictive value in patients with AMI.

In conclusion, we demonstrate that BTL is an independent predictor of all-cause and cardiovascular mortality during the first year post-AMI. We also demonstrate that TL is a tissue-specific biomarker of oxidative stress *in vivo*. At a clinical level, BTL is an easily measurable biomarker that could predict mortality post-AMI, potentially reflecting redox-mediated activation of circulating inflammatory cells.

## Supplementary material

Supplementary material is available at *European Heart Journal* online.

## Funding

The study was funded by the British Heart Foundation (FS/16/15/32047 and PG/13/56/30383 to C.A.), British Heart Foundation Centre of Research Excellence Oxford (RE/08/004 to M.M./C.A.), the National Institute for Health Research Oxford Biomedical Research Centre (C.A./K.C.) and the European Commission (ITN network RADOX to C.A./K.C./F.S.). Open Access funding provided by Oxford’s RCUK Open Access Block Grant.

**Conflict of interst:** none declared.

## Supplementary Material

Supplementary Data
