# Supplementary material for: Predictive value of telomere length on outcome following acute myocardial infarction: evidence for contrasting effects of vascular vs. blood oxidative stress
Source: Eur Heart J. 2017 Apr 24;38(41):3094–104. doi: 10.1093/eurheartj/ehx177 (PMC5837455; doi:10.1093/eurheartj/ehx177)
Supplement: Supplementary Data [file ehx177_supplementary_files_r3.pdf]

Predictive value of telomere length on outcome following acute myocardial infarction: Evidence for contrasting effects of vascular vs. blood oxidative stress

Marios Margaritis[1*], Fabio Sanna[1*], George Lazaros[3], Ioannis Akoumianakis[1], Sheena Patel[1], Alexios S Antonopoulos[1], Chloe Duke[1], Laura Herdman[1], Costas Psarros[1], Evangelos K Oikonomou[1], Cheerag Shirodaria[1] , Mario Petrou[2], Rana Sayeed[2], George Krasopoulos[2], Regent Lee[1], Dimitris Tousoulis[3], Keith M Channon[1], Charalambos Antoniades[1]

[1]: Cardiovascular Medicine Division, University of Oxford, UK

[2]: Department of Cardiac Surgery, John Radcliffe Hospital, Oxford UK

[3]:1st Department of Cardiology, Athens University Medical School, Greece

Running title: "Telomere Length and mortality post-MI"

This work received the Young Investigator Award (1st prize) of the European Society of Cardiology Congress 2015, in the "Ageing and Senescence" category

*Authors equally contributed

Corresponding author:
Charalambos Antoniades MD PhD,
Associate Professor of Cardiovascular Medicine,
Cardiovascular Medicine Division, University of Oxford
John Radcliffe Hospital West Wing Level 6, Headley Way, Oxford OX3 9DU, UK
Tel: +44-1865-228340, Fax: +44-1865-234615
e-mail: antoniad@well.ox.ac.uk

Detailed Methods

Blood sampling and circulating biomarker measurements

Cohort 1 was designed as a prospective outcome study of patients with acute myocardial infarction (AMI), focused on exploring the role of blood telomere length (BTL) and routine plasma biomarkers (such as creatinine, B-type brain natriuretic peptide (BNP) and high sensitivity C-reactive protein (hsCRP)) as predictors of clinical outcome during the first year after the event. Blood samples were collected on hospital admission prior to percutaneous coronary intervention (PCI) for plasma biomarker measurements as part of routine clinical care. Blood samples for BTL were obtained after the patients were consented, typically after they had primary PCI or thrombolysis for ST segment elevation myocardial infarction (STEMI). Follow-up blood samples were obtained thereafter on a daily basis throughout hospitalisation to measure changes in circulating biomarkers. Plasma was used for measurements of troponin I (Abbott Diagnostics, Abbott Park, Illinois) and creatinine by using a colorimetric enzymatic method in a Technicon automatic analyzer RA-1000 (Dade-Behring Marburg GmbH, Marburg, Germany). B-type brain natriuretic peptide (BNP) was also measured by a rapid immunofluorescence assay ([Triage® System for BNP, Biosite diagnostics, La Jolla, CA, USA). hsCRP was measured by a high-sensitivity latex enhanced immunoturbidimetric assay (ADVIA, Bayer HealthCare LLC).

Cohort 2 was used as a mechanistic study to allow for better understanding of the links between BTL, vascular telomere length (VTL) and oxidative stress in the human vascular wall (internal mammary arteries (IMA) and saphenous veins (SV)). Cohort 2 was also used as a source of peripheral blood mononuclear cells (PBMNC) and primary vascular smooth muscle cells (VSMC), used in *ex vivo* cell culture experiments. A more extensive panel of plasma biomarkers was analysed in this cohort, due to sample availability. Blood samples

were obtained before cardiac surgery, after 8h fasting. Blood was also centrifuged at 2000g/4°C for 15 minutes and plasma stored at -80°C for circulating biomarker measurements. Plasma malonyldialdehyde (MDA, a marker of systemic oxidative stress) was measured using the thiobarbituric acid reactive substances (TBARS) fluorometric assay.[1] hsCRP was measured by a high-sensitivity latex enhanced immunoturbidimetric assay (ADVIA, Bayer HealthCare LLC). Interleukin 6 (IL-6) and monocyte chemoattractant protein 1 (MCP-1) were measured using ELISA (R&D systems, U.S.A.). BNP was quantified by a chemiluminescent-microparticle immunoassay (Architect BNP, Abbott, Germany).

Isolation of peripheral blood mononuclear cells (PBMNC)

Blood samples from 128 patients in Cohort 2 were also collected for PBMNC isolation, using gradient centrifugation and accuspin histopaque 1077 (Sigma). More specifically, whole blood was gently poured on the surface of 3mL of histopaque 1077 in 15mL falcon tubes, and the mixture was centrifuged at 400g in room temperature for 30min with zero deceleration to separate mononuclear cells from red blood cells and plasma. The layer containing the PBMNC was then transferred into new tubes and washed with 1X phosphate-buffered saline followed by centrifugation at 250 g for 10 min in room temperature to pellet PBMNCs. This process was repeated twice, and the cell pellets were finally suspended in PBS. Blood was processed immediately after collection in a standardized way to limit any technical variability. PBMNC were snap-frozen in -80°C immediately after isolation until further assayed.

DNA extraction and genotyping

DNA extraction from whole blood: DNA was extracted from whole blood using the QIAamp DNA blood Midi kit (QIAgen, Stanford, CA).

DNA extraction from human vessels: Vascular segments were homogenised in ATL lysis buffer (QIAgen, Stanford, CA); proteinase K (QIAgen, Stanford, CA) was added and the samples were incubated in 56°C for 1 hour with shaking every 15min. Supernatants were collected after centrifugation at 2,000g/10min; DNA was precipitated with ethanol and dissolved in TE buffer (Ambion).

DNA extraction from human VSMC: After trypsinization, VSMC were lysed in lysis buffer (10mM Tris-HCl pH=8, 150mM NaCl, 1mM EDTA and 1% NP-40). DNA was isolated using phenol/chloroform/isoamylalcohol (24:25:1). After centrifugation at 10,000rpm, the upper phase containing the DNA was collected. DNA was precipitated with sodium acetate (3M, pH=5.2) and two volumes of isopropanol, followed by centrifugation at 10,000rpm/30min at 4°C. DNA pellets were re-suspended in 20μL of H_2O.

Genotyping: Genotyping for rs4673 and rs1049255 (functional polymorphisms in the *CYBA* locus encoding for the p22^{phox} subunit of NADPH-oxidases[2, 3]) as well as for rs1041740 (functional polymorphism in the gene encoding for SOD1[4]) and rs4880 (functional polymorphism in the gene encoding for SOD2[5]) were performed using TaqMan probes (Applied Biosystems, Foster City, CA; C_2038_20, C_7516913_10, C_27540738_10, C_8709053_10 respectively) according to manufacturer's conditions.

Measurement of telomere length (TL): Quantitative PCR was used for TL measurement as described by Cawthon *et al.*[6] Custom primer pairs (telg and telc) binding to 5 out of the 6 nucleotides of the TTAGGG repeat sequence of telomeres were used (Invitrogen), the intentional introduction of a mismatch preventing primer-dimer formation. Primer sequences were: 5'ACACTAAGGTTTGGGTTTGGGTTTGGGTTTGGGTTAGTGT3' for telg and 5'TGTTAGGTATCCCTATCCCTATCCCTATCCCTATCCCTAACA3' for telc. Primer

pairs for a single copy gene (albumin, serving as reference gene), were also used. The albumin primer (albu, albd) sequences used are as follows: 5'CGGCGGCGGGCGGCGCGGGCTGGGCGGAAATGCTGCACAGAATCCTTG3' for albu, 5'GCCCGGCCCGCCGCGCCCGTCCCGCCGGAAAAGCATGGTCGCCTGTT3' for albd. SYBR green master mix (Invitrogen) was used for all reactions. Reaction conditions were as follows. Stage 1: 15 min at 95°C. This step serves to denature gDNA and activate DNA polymerase. Stage 2: 2 cycles of 15 s at 94°C, 15 s at 49°C, Stage 3: 32 cycles of 15 s at 94°C, 10 s at 62°C, 15 s at 74°C with signal acquisition, 10 s at 84°C, 15 s at 88°C with signal acquisition. A standard curve was created from serial dilutions of pooled DNA from the cohort. Measurements were performed in duplicates, in 384-well plates on an ABI 7900HT cycler. Interpolated PCR product quantities for telomere length and albumin were expressed relative to the same reference sample of the standard curve. Results are expressed as telomere/single copy gene (T/S) ratio. Duplicates with a SD>0.5 were re-run or excluded from analysis. The inter-assay coefficient of variation for the telomere reaction and for the single copy gene (albumin) reaction were 2.44% and 2.91% respectively. The investigator performing the assay was blinded to all other investigational parameters and patient outcome.

Telomere Restriction Fragment (TRF) Analysis

Telomere length was measured using Telo TAGGG Telomere Length Assay kit (Sigma), according to the instructions provided by the manufacturer. Briefly, 2.5ug of genomic DNA digested with RsaI and HinfI restriction enzymes was loaded in a 0.8% agarose gel and electrophoretically separated for 4h. DNA was then transferred onto nylon membrane by capillary blotting with 20x SSC buffer and fixed to the membrane by UV light for 20 minutes. DNA fragments were then hybridized with a digoxigenin-labelled TAGGG probe (DIG-probe), followed by immunodetection using anti-DIG specific antibody coupled with

alkaline-phosphatase. The probed DNA telomere fragments were finally visualized using a chemiluminescent substrate of alkaline-phosphatase provided with the kit. Total telomere length, expressed as kpb, was finally quantified using TeloTool software (Mathworks).

Assessment of endothelial function

Vascular endothelial function was evaluated non-invasively by measuring the flow-mediated dilatation (FMD) of the brachial artery using ultrasound with a linear array transducer, followed by automated off-line analysis (Vascular Analyser, Medical Imaging Applications LLC), as described.[7] Briefly, brachial artery diameter was recorded before and sixty seconds after a five-minute forearm blood flow occlusion. FMD was defined as the percentile change in vessel diameter after forearm ischaemia, as previously described.[8] This technique has been validated against the vasomotor responses of *ex vivo* preparations of radial arteries to acetylcholine, with excellent correlations[8]. FMD studies were performed by two experienced technicians who were blinded to all other investigational parameteres. The inter-operator variability calculated was 17.4%.

Harvesting and processing of human vessels

IMA and SV samples were harvested during CABG and collected in oxygenated (95% O_2/5% CO_2) ice-cold Krebs Hensleit buffer as described.[9] Each vessel was washed and separated from its adjacent adipose tissue in the lab, under magnification by the same operator. Similar anaesthetics were used in all cases, and each sample was always obtained at the same stage of the operation, to limit between-patients variability.

Superoxide ($O_2^{\cdot -}$) measurements in human vessels

Vascular $O_2^{\cdot -}$ production was measured in fresh, intact vascular segments by lucigenin (Sigma, 5 μmol/L)-enhanced chemiluminescence, as described previously.[10] Briefly, vessels were opened longitudinally to expose the endothelial surface and equilibrated for 20 minutes in oxygenated (95% O_2/5% CO_2) Krebs-HEPES buffer (pH 7.4) at 37°C. The activity of NADPH-oxidases was evaluated by measuring the NADPH (Sigma, 100 μmol/L)-stimulated $O_2^{\cdot -}$.

This technique has been validated by our group against oxidative microfluorescent microtomography using dihydroethidium.[7, 9, 11] The method has also been validated using Vas2870 40μmol/L (an inhibitor of NADPH-oxidases), polyethylene glycol superoxide dismutase 300U/mL (PEG-SOD) and Tiron 10mM (direct scavenger of $O_2^{\cdot -}$) to inhibit the recorded signal for both NADPH-stimulated and unstimulated vascular segments. In those validation experiments, we have observed a (49.4±8.1)% reduction of the resting signal by Vas2870, (81.8±5.9)% reduction by Tiron and (72.7±17.8)% reduction by PEG-SOD. Similarly, we have observed a (53.5±8.3)% reduction of the NADPH-stimulated signal by Vas2870, (80.9±13.3)% reduction by Tiron and (72.8±4.0)% reduction by PEG-SOD.

NADPH-oxidases activity in PBMNC

$O_2^{\cdot -}$ production in PBMNC was measured by a lucigenin-enhanced chemiluminescence assay[12], using lucigenin 5 μmol/L chemiluminescence. More specifically, the volume required to load 10^6 cells was determined, and the samples were subjected to three freeze-thaw cycles for cell lysis. Krebs-HEPES buffer and lucigenin were added into a luminometry vial to record the background signal before adding the appropriate sample volume. NADPH-

stimulated $O_2^{\cdot -}$ was used as final readout. Results are finally expressed as RLUs/sec./10^3 cells. Each sample was used only once and the leftover was discarded.

The specificity of this assay has been validated by inhibiting the recorded NADPH-stimulated signal with specific inhibitors. In particular, addition of Vas2870 40μmol/L, an inhibitor of NADPH-oxidases, results in (83.8±7.8)% inhibition of the signal. Similarly, superoxide dismutase (SOD) 300U/mL and Tiron 10mM (a direct scavenger of $O_2^{\cdot -}$) are able to inhibit the NADPH-stimulated PBMNC by (75.3±4.5)% and (86.2±9.1)% respectively.

Cell culture experiments

To isolate VSMC, SV explants were washed in PBS, adventitia and intima layers were removed and the tissue was digested with trypsin for 10 minutes at 37°C. The digested tissue was diced into 1mm^2 sections and cultured in DMEM+20% FBS. VSMC were then split in a new flask before reaching confluence and grown in Media 231 supplemented with smooth muscle growing supplement (SMGS) (Life Technologies Ltd). VSMC were used for experiments after the second splitting passage, around 6 weeks after the SV was explanted.VSMC were then treated with AngII for 30 days (on a 24h on/off pattern). The processing of VSMC for a total of 72 days before any readout was recorded allowed for a washout of any medication the patients were being treated with, eliminating any potential confounding effects on the results of the cell culture experiments.

Superoxide measurement in VSMC: $O_2^{\cdot -}$ production in VSMC was measured using lucigenin-enhanced chemiluminescence. Briefly, 1x10^5 VSMC were scraped in ice-cold buffer (KH_2PO_4 50mM, EGTA 1mM, sucrose 150mM, pH=7), then lysed in an ultrasound bath. Results were normalised to protein content.

Comet assay

DNA damage was evaluated by using the comet assay (CometAssay kit, Trevigen, Maryland, United States). Briefly, $1x10^5$ VSMCs were suspended in 50uL of PBS plus 500uL of melted low-melting agarose, mixed thoroughly and 50uL finally distributed on a glass coverslip. Samples were then processed according to the instructions provided by the manufacturer. DNA damage was expressed as percentage of DNA in the comet tail, as determined by OpenComet software.

Antioxidant measurements

Plasma total antioxidant capacity (TAC) was measured colorimetrically using a commercially available kit (Cell Biolabs, San Diego, CA). Plasma and tissue superoxide dismutase (SOD) activity was measured colorimetrically using a commercially available kit (Abcam, Cambridge, UK).

Supplementary tables

Table S1: Demographic characteristics of patients in Cohort 1

	**Short BTL (T/S ratio <0.916)**	**Long BTL (T/S ratio ≥0.916)**	**P value**
n	126	164	-
Age (years)	63.2±12.6	62.8±12.8	0.79
Male gender n, (%)	109 (86.5)	138 (84.1)	0.61
Hypertension n, (%)	79 (62.7)	99 (60.4)	0.71
Hyperlipidaemia n, (%)	72 (57.1)	97 (59.1)	0.80
T2DM n, (%)	35 (27.8)	45 (27.4)	0.89
Active smoking n, (%)	80 (63.4)	101 (61.5)	0.90
BMI (kg/m^2)	26.5±2.6	27.0±2.5	0.80
**Type of AMI n, (%)**			
STEMI	70 (55.5)	94 (57.3)	0.81
NSTEMI	56 (44.5)	70 (42.7)	0.81
**Management of STEMI n, (%)**			
Primary PCI	86 (68.2)	110 (67.1)	0.89
Thrombolysis & PCI within 24h	40 (31.8)	54 (32.9)	0.89
**Medication n, (%)**			
Aspirin	65 (51.8)	87 (52.8)	0.81
Clopidogrel	3 (2.6)	5 (3.2)	1.00
ACEi/ARBs	83 (66.1)	112 (68.2)	0.71
Statins	69 (54.5)	98 (60.0)	0.40
B-blockers	88 (69.7)	121 (73.6)	0.51
CCB	27 (21.2)	28(17.2)	0.37
Insulin/Oral hypoglycaemic	28 (22.4)	35 (21.6)	1.00
**SOD genetic polymorphisms n, (%)**			
0 alleles	8 (6.4)	12 (7.3)	0.82
1 allele	46 (36.4)	60 (36.7)	1.00
2 alleles	42 (33.6)	51 (31.3)	0.71
3 alleles	25 (20.0)	34 (20.7)	0.88
4 alleles	5 (3.6)	7 (4.0)	1.00

BTL: Blood telomere length; T2DM: Type 2 diabetes mellitus; BMI: body mass index; STEMI: ST segment elevation myocardial infarction; NSTEMI: Non-STEMI; PCI: percutaneous coronary intervention; ACEi: Angiotensin converting enzyme inhibitors; ARBs: Angiotensin receptor blockers; CCB: Calcium channel blockers; SOD: Superoxide dismutase; data presented as mean±standard deviation (SD). There was no significant difference in any of the examined variables across the two groups. P values derived from unpaired t-test for continuous variables and Fisher's exact test for categorical variables.

Table S2: Demographic characteristics of patients from Cohort 2 that participated in the individual *ex vivo* experiments

	PBMNC	VTL in IMA	VTL in SV	Cell culture experiments	P value
Participants (n)	128	32	24	6	
Age (years)	63.8±11.3	67.1±7.4	68.6±8.8	71.3±4.4	0.05
Male gender n, (%)	95 (74.2)	25 (77.4)	21 (87.0)	4 (66.6)	0.51
Hypertension n, (%)	99 (77.2)	23 (73.3)	18 (75.0)	4 (66.6)	0.87
Hyperlipidaemia n, (%)	75 (58.6)	21 (65.6)	15 (63.6)	6 (100)	0.22
T2DM n, (%)	28 (21.9)	11 (34.4)	7 (30.4)	3 (50)	0.24
Active smoking n, (%)	22 (17.2)	4 (11.1)	3 (14.3)	0 (0)	0.62
BMI (kg/m^2)	28.8±4.5	27.4±4.3	27.6±6.2	31.3±5.0	0.16
**Medication n, (%)**					
Aspirin	102 (79.7)	30 (93.8)	20 (81.8)	5 (83.3)	0.32
Clopidogrel	44 (34.4)	4 (12.5)	4 (18.2)	0 (0)	0.02
ACEi/ARBs	91 (71.1)	23 (71.5)	15 (62.2)	2 (33.3)	0.22
Statins	98 (76.6)	31(96.9)*	22 (90.9)	6 (100)	0.02
β blockers	98 (70.3)	31 (78.1)	22 (77.3)	6 (100)	0.31
CCB	28 (21.9)	14 (43.8)	8 (31.8)	1 (16.6)	0.07
Anti-diabetic treatment	21 (16.4)	8 (25)	5 (20.8)	3 (50.0)	0.08

PBMNC: Peripheral blood mononuclear cells; VTL: vascular telomere length; IMA: Internal mammary arteries; SV: Saphenous veins; T2DM: Type 2 diabetes mellitus; BMI: body mass index; ACEi: Angiotensin converting enzyme inhibitors; ARBs: Angiotensin receptor blockers; CCB: Calcium channel blockers; data presented as mean±standard deviation (SD). P-values referred to one-way ANOVA. Comparisons between all groups were performed using unpaired t-test or Fisher's exact test, and P values were corrected for multiple testings using Bonferoni post-hoc correction; *P<0.05 vs PBMNC; There were no other significant differences between the groups.

Supplementary figures and legends

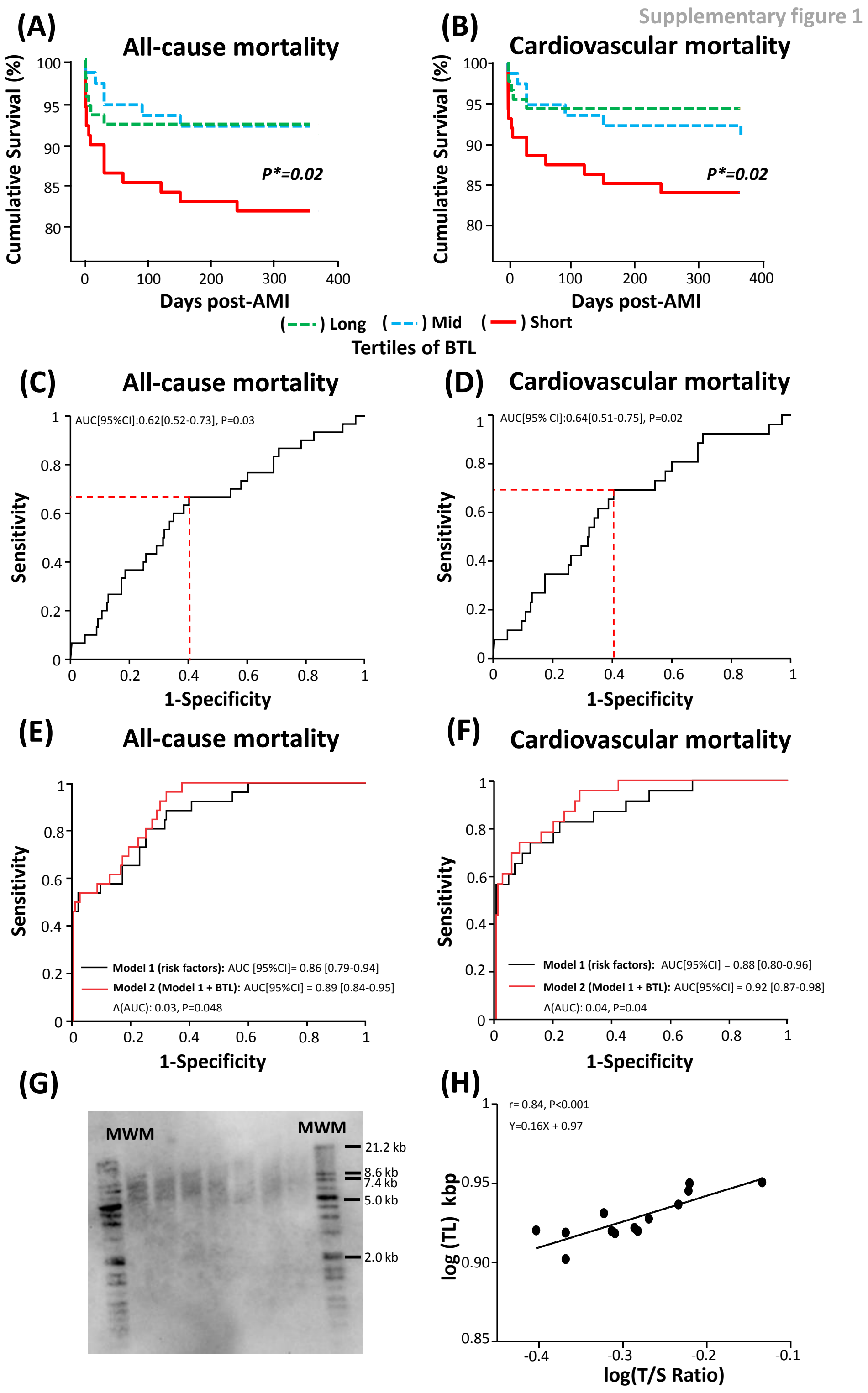

Figure S1. Receiver operating characteristic (ROC) curves for all-cause and cardiovascular mortality: <u>In Kaplan-Meier analysis, short blood telomere length (BTL) was associated with a significantly higher risk of all-cause (panel A) and cardiovascular mortality (panel B) compared to the combined mid and long BTL groups in</u>

patients followed up to 1 year following acute myocardial infarction (AMI). Interestingly, no significant difference was found between the mid and long BTL tertiles for either endpoint (P=0.86 for all-cause and P=0.45 for cardiovascular mortality), suggesting the presence of a threshold effect. Next, Receiver Operating Characteristic (ROC) analysis was performed to define a BTL cut-off for the prediction of all-cause (panel C) and cardiovascular mortality (panel D). This analysis indicated that a BTL cut-off value of 0.916 would predict all-cause mortality with 66.7% sensitivity & 59.7% specificity, as well as cardiovascular mortality with 69.2% sensitivity & 59.6% specificity. To examine the incremental prognostic value of BTL using this cut-off, beyond established risk factors, we used C-statistics to compare the changes of the ROC curves after adding this new biomarker into a model that includes risk factors and standard biomarkers used in routine clinical practice. We observed a small but significant improvement of the predictive value of both the model of all-cause (panel E) and cardiovascular mortality (panel F). To determine the absolute telomere lenth that corresponds to the cut-off T/S=0.916, we performed the telomere restriction fragment (TRF) analysis in DNA from 13 patients (panel G), and we correlated the results with the T/S assay for BTL (Panel H). Using regression anlysis, we estimated that the telomere length corresponding to the T/S cut-off was 9.29 kbp. Model 1: the optimum model that includes all known clinical risk factors (age, gender, hypertension, dyslipidemia, diabetes, smoking, type of MI, number of diseased vessels), circulating biomarkers (troponin, BNP, CRP, plasma lipid levels, and peak troponin) and medication (antiplatelet treatment, beta-blockers, ACEi/ARBs and statins); Model 2: Model 1 + BTL >0.916; *comparisons between long BTL tertile vs the other 2 tertiles using the Breslow test.

Supplementary figure 2

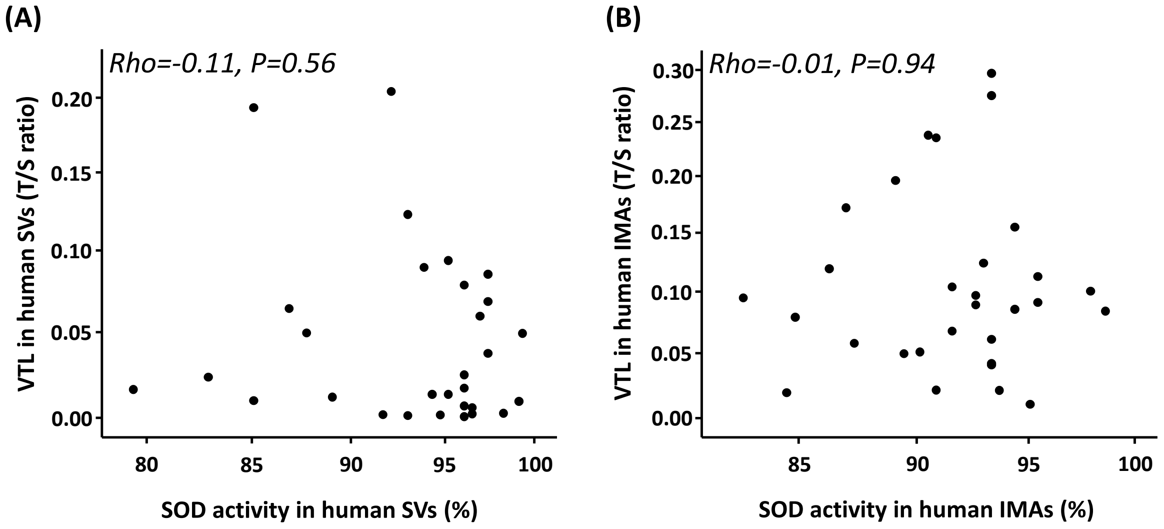

Figure S2. Correlation between vascular telomere length (VTL) and superoxide dismutase (SOD) activity in human vessels: In order to elucidate the role of endogenous anti-oxidant defences in the regulation of VTL, we evaluated the activity of SOD in human saphenous vein (SV) and internal mammary artery (IMA) segments from 30 patients, and correlated it with VTL measurements in the same vessels. No significant association was observed between SOD activity and VTL either in SV (panel A) or IMA (panel B). P-values calculated by Spearman's rank correlation.

Supplementary figure 3

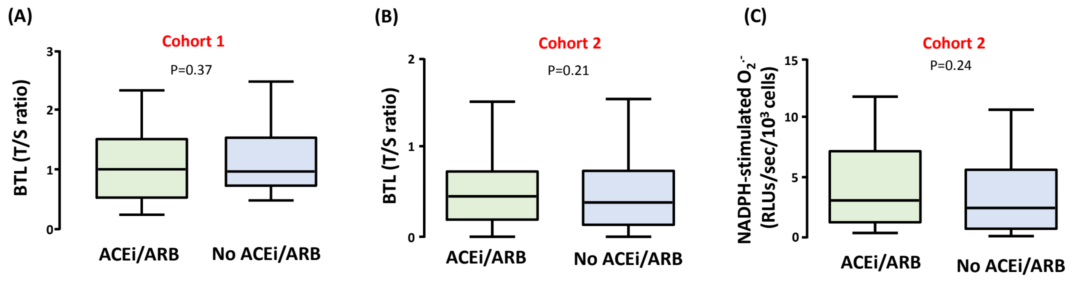

Figure S3: Effect of angiontensin converting enzyme inhibitor (ACEi) or angiotensin receptor blocker (ARB) treatment on blood telomere length (BTL) and superoxide ($O_2^{\cdot-}$) production from peripheral blood mononuclear cells (PBMNC): No significant association was observed between ACEi/ARB anti-hypertensive treatment and BTL in either Cohort 1 (panel A) or Cohort 2 (panel B). Furthermore, in Cohort 2, ACEi/ARB medication was not related with $O_2^{\cdot-}$ production in PBMNC (C). Values presented as median[25th-75th percentiles]. P-values calculated by Mann Whitney U tests.

Supplementary figure 4

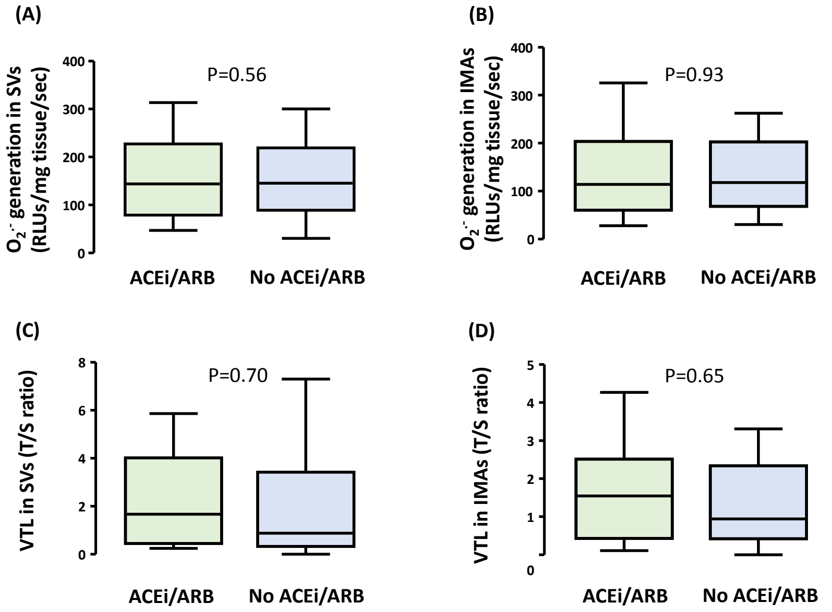

Figure S4: Effect of angiontensin converting enzyme inhibitor (ACEi) or angiotensin receptor blocker (ARB) treatment on vascular telomere length (VTL) and vascular superoxide ($O_2^{\cdot -}$) generation in human vessels: No significant association was observed between ACEi/ARB anti-hypertensive treatment and $O_2^{\cdot -}$ production in either saphenous vein (SV) segments (panel A) or internal mammary artery (IMA) segments (panel B). Similarly, ACEi/ARB treatment was not correlated with VTL in SVs (panel C) or IMAs (panel D). Values presented as median[25th-75th percentiles]. P-values calculated by Mann Whitney U tests.

References

1. Antoniades C, Bakogiannis C, Tousoulis D, Reilly S, Zhang MH, Paschalis A, Antonopoulos AS, Demosthenous M, Miliou A, Psarros C, Marinou K, Sfyras N, Economopoulos G, Casadei B, Channon KM, Stefanadis C. Preoperative atorvastatin treatment in CABG patients rapidly improves vein graft redox state by inhibition of Rac1 and NADPH-oxidase activity. Circulation 2010;**122**(11 Suppl):S66-73.
2. Guzik TJ, West NE, Black E, McDonald D, Ratnatunga C, Pillai R, Channon KM. Functional effect of the C242T polymorphism in the NAD(P)H oxidase p22phox gene on vascular superoxide production in atherosclerosis. Circulation 2000;**102**(15):1744-7.
3. Inoue N, Kawashima S, Kanazawa K, Yamada S, Akita H, Yokoyama M. Polymorphism of the NADH/NADPH oxidase p22 phox gene in patients with coronary artery disease. Circulation 1998;**97**(2):135-7.
4. Heslop CL, Tebbutt SJ, Podder M, Ruan J, Hill JS. Combined polymorphisms in oxidative stress genes predict coronary artery disease and oxidative stress in coronary angiography patients. Annals of human genetics 2012;**76**(6):435-47.
5. Jones DA, Prior SL, Tang TS, Bain SC, Hurel SJ, Humphries SE, Stephens JW. Association between the rs4880 superoxide dismutase 2 (C>T) gene variant and coronary heart disease in diabetes mellitus. Diabetes research and clinical practice 2010;**90**(2):196-201.
6. Cawthon RM. Telomere length measurement by a novel monochrome multiplex quantitative PCR method. Nucleic acids research 2009;**37**(3):e21.
7. Margaritis M, Antonopoulos AS, Digby J, Lee R, Reilly S, Coutinho P, Shirodaria C, Sayeed R, Petrou M, De Silva R, Jalilzadeh S, Demosthenous M, Bakogiannis C, Tousoulis D, Stefanadis C, Choudhury RP, Casadei B, Channon KM, Antoniades C. Interactions between vascular wall and perivascular adipose tissue reveal novel roles for adiponectin in

the regulation of endothelial nitric oxide synthase function in human vessels. Circulation 2013;**127**(22):2209-21.

8. Antoniades C, Mussa S, Shirodaria C, Lee J, Diesch J, Taggart DP, Channon KM, Leeson P. Relation of preoperative radial artery flow-mediated dilatation to nitric oxide bioavailability in radial artery grafts used in off-pump coronary artery bypass grafting. The American journal of cardiology 2009;**103**(2):216-20.

9. Antonopoulos AS, Margaritis M, Coutinho P, Shirodaria C, Psarros C, Herdman L, Sanna F, De Silva R, Petrou M, Sayeed R, Krasopoulos G, Lee R, Digby J, Reilly S, Bakogiannis C, Tousoulis D, Kessler B, Casadei B, Channon KM, Antoniades C. Adiponectin as a link between type 2 diabetes and vascular NADPH oxidase activity in the human arterial wall: the regulatory role of perivascular adipose tissue. Diabetes 2015;**64**(6):2207-19.

10. Antoniades C, Bakogiannis C, Leeson P, Guzik TJ, Zhang MH, Tousoulis D, Antonopoulos AS, Demosthenous M, Marinou K, Hale A, Paschalis A, Psarros C, Triantafyllou C, Bendall J, Casadei B, Stefanadis C, Channon KM. Rapid, direct effects of statin treatment on arterial redox state and nitric oxide bioavailability in human atherosclerosis via tetrahydrobiopterin-mediated endothelial nitric oxide synthase coupling. Circulation 2011;**124**(3):335-45.

11. Antonopoulos AS, Margaritis M, Verheule S, Recalde A, Sanna F, Herdman L, Psarros C, Nasrallah H, Coutinho P, Akoumianakis I, Brewer AC, Sayeed R, Krasopoulos G, Petrou M, Tarun A, Tousoulis D, Shah AM, Casadei B, Channon KM, Antoniades C. Mutual Regulation of Epicardial Adipose Tissue and Myocardial Redox State by PPAR-gamma/Adiponectin Signalling. Circulation research 2016;**118**(5):842-55.

12. Van Nerom A, Desmidt M, Ducatelle R, Haesebrouck F. Lucigenin- and luminol-enhanced chemiluminescence in turkey monocytes. Journal of bioluminescence and chemiluminescence 1997;**12**(4):207-14.
